# Early Events, Kinetic Intermediates and the Mechanism of Protein Folding in Cytochrome *c*

**DOI:** 10.3390/ijms10041476

**Published:** 2009-04-01

**Authors:** Robert A. Goldbeck, Eefei Chen, David S. Kliger

**Affiliations:** Department of Chemistry and Biochemistry, University of California, Santa Cruz, California 95046, USA; E-Mails: chen@chemistry.ucsc.edu (E.C.); kliger@chemistry.ucsc.edu (D.K.)

**Keywords:** Collapsed intermediate, secondary structure formation, disordered tertiary structure, conformational diffusion, unfolded chains, molten globule, heme misligation, time-resolved spectroscopy, far-UV circular dichroism, magnetic circular dichroism, Trp59 fluorescence, amide hydrogen exchange, small-angle X-ray scattering, thermophiles, three-state pathway

## Abstract

Kinetic studies of the early events in cytochrome *c* folding are reviewed with a focus on the evidence for folding intermediates on the submillisecond timescale. Evidence from time-resolved absorption, circular dichroism, magnetic circular dichroism, fluorescence energy and electron transfer, small-angle X-ray scattering and amide hydrogen exchange studies on the *t* ≤ 1 ms timescale reveals a picture of cytochrome *c* folding that starts with the ~ 1-μs conformational diffusion dynamics of the unfolded chains. A fractional population of the unfolded chains collapses on the 1 – 100 μs timescale to a compact intermediate I_C_ containing some native-like secondary structure. Although the existence and nature of I_C_ as a discrete folding intermediate remains controversial, there is extensive high time-resolution kinetic evidence for the rapid formation of I_C_ as a true intermediate, i.e., a metastable state separated from the unfolded state by a discrete free energy barrier. Final folding to the native state takes place on millisecond and longer timescales, depending on the presence of kinetic traps such as heme misligation and proline mis-isomerization. The high folding rates observed in equilibrium molten globule models suggest that I_C_ may be a productive folding intermediate. Whether it is an obligatory step on the pathway to the high free energy barrier associated with millisecond timescale folding to the native state, however, remains to be determined.

## 1. Introduction

**Figure 1.**
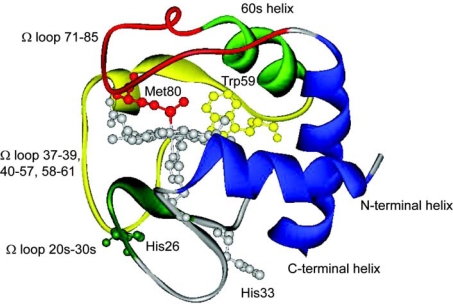
Drawing of the native structure of horse heart cytochrome *c* showing the heme coordinated by His18 and Met80, the locations of His26 and His33 (which can bind to heme under denaturing conditions), and Trp59 (PDB 1hrc). The foldons, discrete units of secondary and tertiary structure that unfold sequentially in the order red, yellow, green, and blue, are shown as identified by Bai *et al.* [16] (see text).

The small globular protein cytochrome *c* has played a large role in studies of the earliest events and intermediates in protein folding. Its outsized role has stemmed in part from its availability, robustness, and relatively simple single domain structure, and in part from the unique presence of a covalently bound heme prosthetic group. The stability of its folded state is further linked to the heme moiety by coordination bonds with His18 and Met80 at the iron atom’s axial fifth and sixth coordination sites (Figure 1). The coupling of heme oxidation and ligation states with folding driving force has permitted the development of laser photo-initiation methods to conveniently trigger folding on the nanosecond timescale [1–3]. The electronic properties of the heme chromophore also provide convenient spectroscopic and energy transfer probes of folding dynamics for kinetic studies, such as the intense absorption bands of the heme Soret spectral region (400 – 450 nm) and the distance-dependent fluorescent energy transfer from Trp59 to the heme. On the other hand, the presence of the heme moiety may itself add complexity to the folding dynamics, as the protein can lose its native Met80 coordination under denaturing conditions and bond with nonnative ligands, e.g., His33 or H26 in horse cyt *c* [4,5].

**Figure 2.**
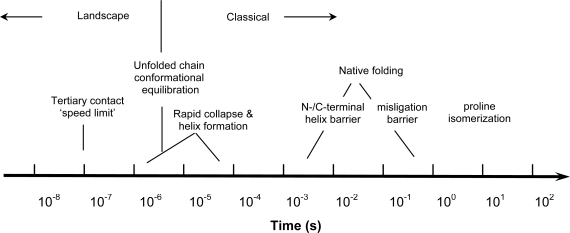
Characteristic timescales of dynamical processes during the folding of cytochrome *c*.

The observed folding dynamics can extend from the hundreds of nanoseconds timescale for initial helix formation [6], through the millisecond acquisition of essentially native secondary and tertiary structure, to the final isomerization of proline bonds on the timescale of seconds (Figure 2) [7]. The folding kinetics of cyt *c* over this ~ 8 orders of magnitude in time can depend qualitatively (number of intermediate states) and quantitatively (rate constants) on the ligation state of the heme (e.g., complexation with exogenous ligands) and on its oxidation state, as well as on the denaturant concentration, pH, and temperature [7]. The result has been a rich array of studies as the folding kinetics of cyt *c* are explored with various combinations of protein and solvent conditions, spectroscopic probes and degrees of time resolution. We review here many of these kinetic studies with a particular eye to the earliest processes, i.e. those on the nanosecond to microsecond timescales, which have become increasingly accessible to experiment over the past ~ 15 years. The existence and nature of these intermediates have been the subject of considerable controversy. We also discuss their import to understanding the cooperative mechanism leading to the appearance of the native fold on the 1 ms and longer timescales. We first briefly survey the kinetics of the late folding processes in cyt *c* in order to set the context before considering the beginning of the folding reaction coordinate and the first dynamic events arising from the unfolded state. The reader is also referred to the earlier review by Winkler, which provides a somewhat broader survey of experimental and theoretical results for cyt *c* folding dynamics [8].

## 2. Late Folding Processes (*t* > 1 ms)

Folding to the native state proceeds on the millisecond and slower timescale in cyt *c*. The number and amplitude of the exponential processes observed in kinetic studies on this timescale depends on the folding conditions used [7]. At neutral pH, the folding process with the largest amplitude observed in ferricytochrome *c* has a time constant of 240 ms (70% amplitude), but minor components are detected with time constants of 10 ms (15%) and 13 s (15%) (in 1.75 M GuHCl). The slowest time constant observed is characteristic of proline isomerization. The largest amplitude process involves folding that is concomitant with the displacement of a histidine residue by Met80 at the heme axial site. Thus, adding imidazole or lowering the pH to 5 eliminates the 240-ms component and shifts the reaction amplitude to the 10-ms component, which represents the folding rate unimpeded by histidine misligation. (The protonation of nonnative histidine ligands below pH ≈ 6 blocks their binding to the heme. Similarly, exogenous imidazole displaces histidine at the sixth axial heme site. Its off rate being much faster than that of a histidine residue attached to the protein backbone, it is not rate limiting in cyt *c* folding.) The amplitude of the proline isomerization process is also much smaller in the presence of exogenous heme ligands. These findings suggest that the fastest of the millisecond timescale processes may be considered the most fundamental to understanding protein folding in that it appears to be independent of the histidine-misligation kinetic trap peculiar to cyt *c* [9].

A pulsed amide-hydrogen exchange (HX) study also observed similar time constants for three folding rate processes in stopped-flow denaturant dilution experiments [10]. Time constants of ~ 10 ms, ~ 100 ms, and ~ 10 s were found to characterize the acquisition of increased HX protection factors at various residues during folding after a GuHCl-jump of 4.2 → 0.7 M (pH 6.2, 10 °C). The HX data were interpreted in terms of a kinetic intermediate formed at about 10 ms with N- and C-terminal helical structures, but with no 60’s and 70’s helix or stable tertiary structure present. At ~ 100 ms, about half the molecules acquired stable tertiary structure. The final development of native structure proceeded with a 10-s time constant. Later pulsed HX studies under somewhat faster folding conditions (0.23 M GuHCl, pH 6.0, 10 °C) looked in more detail at the species trapped at ~ 100 ms by heme misligation in the wild type protein and by N-terminal heme misligation and other barriers in the double histidine mutant H33N/H26N [11,12]. The general picture that emerges from the pulsed HX studies is that the ~ 10-ms barrier represents formation of the N-terminal and C-terminal helices (the highest energy “foldon” identified in native state HX studies discussed below). Protein chains that are not trapped by heme misligation or other barriers then fold quickly to the native state while the remaining chains must wait (with their N-/C-terminal helices already formed) to pass over an additional rate-limiting barrier to form the remaining native structure.

The millisecond folding rate constants generally follow a linear free energy relation (LFER) between activation free energy and driving force, at least for denaturant concentrations not too far away from the *C*_m_. Thus, the increased driving force at low denaturant concentrations tends to increase the observed rate relative to higher denaturant concentrations. The LFER parameter *m*^‡^/*m*, where *m* and *m*^‡^ are the GuHCl concentration dependences of the equilibrium folding and activation free energies, respectively, is 0.4 for cyt *c*^III^ at low pH and 0.45 for cyt *c*^II^ at neutral pH [13,14]. From the Hammond postulate [15], which posits that this parameter can be interpreted as the location of the transition state (TS) along the reaction coordinate, the similarity of the two values suggests that neither heme misligation nor heme iron oxidation state greatly affect the nature of the transition state, which occurs relatively early on the folding reaction coordinate [14].

Native state HX measurements have detected discrete units of cooperatively unfolding structure, foldons, that are reached by transient excursions from the equilibrium folded state [16]. Bai *et al.* inferred from native-state HX GuHCl isotherms a sequential pathway for unfolding comprising the following equilibrium intermediates in which the indicated additional increment of secondary and tertiary structure is cooperatively unfolded in order of increasing free energy: Ω loop for residues 70 – 85, ΔG_o_^u^ = 6.0 kcal/mol; Ω loop for residues 36 – 61, ΔG_o_^u^ = 7.4 kcal/mol; 60’s helix (residues 60 – 69) and Ω loop for residues 25 – 30, ΔG_o_^u^ = 10.0 kcal/mol; and N-/C-terminal helices (residues 2 – 14 and 87 – 104), i.e., the fully unfolded state, ΔG_o_^u^ = 12.8 kcal/mol (energies extrapolated to [GuHCl] = 0 M, native state = zero of energy). (Residues 40 – 57 were later identified by Krishna *et al.* as a separate foldon with ΔG_o_^u^ = 5.0 kcal/mol in cyt *c*^III^ [17].)

It has been further suggested that the reverse of this unfolding pathway represents an obligatory kinetic pathway for the sequential folding of the same discrete units of native structure, i.e., the foldons [18]. The rate-limiting step in this folding pathway is either the formation of the highest energy foldon (N-/C-terminal helices) or, in the presence of an additional barrier from heme misligation, a later metastable intermediate, e.g., the formation of the 60’s helix and residue 25 – 30 loop. This placement of the largest activation barrier at or near the beginning of the pathway is required by the fact that sequentially folded intermediates like those just described have remained largely hidden from observation in cyt *c*. The pulsed HX results mentioned above suggest that the time constant for passage over this barrier is ~ 10 ms. A more controversial corollary of this scenario is the proposal that no other kinetic folding intermediates exist between U, the fully denatured state, and the free energy barrier associated by the HX evidence with N-/C-terminal helix formation [19]. In particular, this view implies that the timescales for cooperative molecular collapse to a state with near-native compaction and for formation of elements of native secondary structure can proceed no faster than ~ 1 – 10 ms in cyt *c*. This contention is discussed further below after considering the experimental evidence for molecular collapse and helix formation processes on the microsecond timescale.

## 3. The Nature of the Unfolded State

It is widely appreciated that chemical denaturants such as guanidine hydrochloride (GuHCl) may induce cooperative transitions to unfolded states in which the protein chains contain residual structure beyond that expected from a random polymer [20 and references therein]. A small-angle X-ray scattering (SAXS) study of ferricytochrome *c* denatured in GuHCl found evidence for at least two equilibrium subensembles of unfolded chain conformations with distinct dependences on denaturant concentration, denoted U_1_ and U_2_ [21]. U_1_, the subensemble that appears first as the GuHCl concentration is raised past the ~ 2.7-M *C*_m_ for unfolding, contains a significant amount of residual nonglobular structure corresponding to nonspecific hydrophobic interactions. Further increasing the denaturant concentration drives U_1_ to the more random coil structure U_2_ with a halfway point of ~ 3.4 M. The radius of gyration, R_g_, of the unfolded states was significantly more extended (30 Å) than that of the native state (14 Å), as expected, although this study did not detect a difference in R_g_ between U_1_ and U_2_. The components U_1_ and U_2_ probably each represented in turn an average of underlying unfolded states that transform with little residue-residue cooperativity to more extended states with increasing denaturant, as suggested by the gradual denaturant concentration dependence of the third singular value decomposition (SVD) component of the SAXS data in the unfolded region.

A more detailed look at the unfolded state was provided by a FET study of GuHCl-denatured yeast iso-1 cyt *c*^III^ using introduced Dns fluorophores as a probe of residue-heme distance at various locations in the protein sequence [20]. The distributions of distances found were more complex and implied a much more heterogeneous mixture of compact and extended structures than would be expected from a random polymer. This heterogeneity was found to be independent of heme misligation (e.g., insensitive to exogenous imidazole-heme binding) and may have been caused at least in part by hydrophobic interactions between the polypeptide chain and the heme. Consistent with the SAXS results, the population of extended structures increased only gradually at higher concentrations of GuHCl, such that high denaturant concentrations did not lead to full elimination of population in the more compact end of the size distribution: 60% of the Dns(C50)-iso-1-cyt *c* molecules were in more compact conformations (mean Dns-heme distance of ~ 30 Å, as compared with ≥ ~ 40 Å in the more extended conformations and 23 Å when folded) at 2.7 M GuHCl (well above the *C*_m_ of 1.3 M for this protein), whereas at 4.4 M GuHCl a 30% population of relatively compact species still remained. HX measurements have also detected residual structure in ferricytochrome *c* denatured in GuHCl [16,22]. The amide protons of Cys14, Ala15, and His18 remain protected from hydrogen exchange at high denaturant concentrations, suggesting persistent residual structure near the points where the heme group is covalently bound to the polypeptide chain.

**Figure 3.**
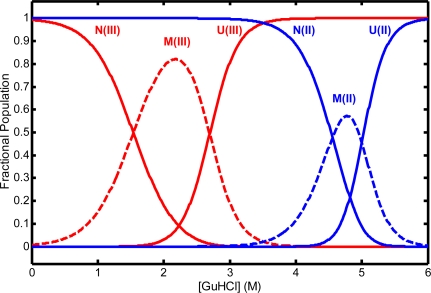
Equilibrium populations of native (N), molten globule (M), and unfolded (U) states of Fe^II^ (blue lines) and Fe^III^ (red lines) cyt *c* vs. GuHCl concentration, calculated from the thermodynamic data of Thomas *et al.*, pH 6.5, 40 °C [24].

As noted above, the possibility of nonnative heme coordination in denatured states can impose constraints on the unfolded chain conformations that may influence folding processes. At near-neutral pH, chemically denatured equine cyt *c*^III^ has predominantly His18-Fe-His33 heme coordination (~ 80%) and a minor His18-Fe-His26 component [23]. Bis-histidine coordination is also found in denatured ferrocytochrome *c* [1,24]. In the H26-coordinated form, the polypeptide backbone loop formed between His18 and His26 by their simultaneous binding to the heme iron atom contains only 9 residues, which must wrap around the heme disk. The greater strain of this arrangement may account for its lower population than the His18-Fe-His33 component. Lowering the pH below the pK of a solvent-exposed histidine residue, ~ 6, or adding exogenous imidazole both block binding of a second histidine ligand to heme. The GuHCl-induced unfolding of cyt *c* is a three-state process, N ⇔ M ⇔ U, passing through an equilibrium molten globule intermediate that has been detected in both oxidation states of the protein (Figure 3) [24].

Loss of native heme coordination and perturbation of tertiary structure near the heme were detected in M by heme band MCD and CD. Note that because this intermediate is populated near what has often been taken as the nominal C_m_ for N → U unfolding, kinetic studies that trigger folding in samples prepared under weak-folding denaturant conditions (1.5 – 2.5 and 4 – 5 M GuHCl in oxidized and reduced cyt *c*, respectively) may also trigger significant amounts of M → N folding. Similarly, samples prepared under strongly denaturing conditions and jumped to weak-folding conditions may also undergo some U → M folding, as well as U → N.

## 4. The Dynamics of the Unfolded State

The dynamics of loop formation in the unfolded chains have been of great interest with respect to various “speed limits” for folding. Hagen *et al.* [25] monitored the photodissociation of the CO complex of ferrocytochrome *c* in 4.6 M GuHCl with time-resolved optical absorption (TROA) spectroscopy in the Soret band to examine the rate at which Met80 rebinding to the vacated axial heme site forms a 65-residue loop between the methionine residue and His18. (Due in part to stronger heme-residue coordination bonds and reduced electrostatic repulsion in the reduced state, its GuHCl *C*_m_ of 4.9 M is 2.2 M higher than in the oxidized form [24]. Exogenous CO can displace Met80 as a heme ligand to form a complex with a lowered *C*_m_ that is largely unfolded in 4.6 M GuHCl, allowing CO photolysis to be used as a folding trigger [1].) From their assignment of the microscopic rate constant for methionine binding after CO photolysis, they inferred a loop-closure time of 40 μs. They then used the N^3/2^ length dependence of intrachain diffusion times predicted by simple polymer theory to extrapolate from N = 65 to N = 10, the size of the most probable tertiary-contact loops expected to form in folding [26]. Since folding to the native state cannot proceed faster than the rate at which tertiary contacts are formed, the extrapolated rate of (1 μs)^−1^ was suggested to represent an ultimate speed limit for folding. Goldbeck *et al.* studied this system with time-resolved magnetic circular dichroism (TRMCD) spectroscopy and found qualitative evidence in the heme-residue binding kinetics that finite intrachain diffusion prevented complete equilibration of the unfolded chain conformers on this timescale (from 2 μs for methionine binding to 50 μs for histidine binding) [27]. Similarly, heterogeneous folding kinetics observed in the photo-ET-triggered folding of ferrocytochrome *c* suggested incomplete equilibration of the unfolded chains on the timescale of the bimolecular reduction process, τ_1/2_ ≈ 5 μs [28]. Chang *et al.* measured electron transfer (ET) rates in unfolded cyt *c* modified by substituting the heme with an excited-state electron donor, Zn-porphyrin, and attaching an electron acceptor, Ru(NH_3_)_5_^3+^, to His33 [29]. The observed ET rates implied a 15-residue loop closure time of 250 ns. Extrapolating this observation to N = 10 gave a folding speed limit of (100 ns)^−1^. A similar extrapolation to N = 65 gives 2 μs as a rough estimate for the conformational equilibration time of the unfolded chain ensembles with respect to the loop closures involved in heme-residue binding and, ultimately, formation of the native state. The latter time constant was measured more directly by Abel *et al.*, who applied a kinetic model explicitly accounting for the effect of unfolded conformational exchange on the residue binding kinetics to TRMCD data obtained from CO photolysis experiments performed on several histidine variants of cyt *c* [30]. That study found a conformational equilibration time of ~ 3 μs, in agreement with the extrapolation from the small-loop data of Chang *et al.* The latter estimates of faster intrachain diffusion rates in cyt *c* are also similar to those measured in non-folding polypeptides when adjusted for chain length [31–33]. This agreement tends to allay concerns raised by the earlier evidence that the unfolded chain dynamics of cyt *c* may be anomalously slow [8]. Currents estimates of an ultimate speed limit for folding based on N ≈ 10 loop formation thus converge at ~ (100 ns)^−1^.

The conformational equilibration time of unfolded cyt *c* also appears to be very similar in magnitude to the “molecular timescale” of 2 μs inferred by Yang and Gruebele for a non-heme fivehelix bundle protein of similar size, λ_6–85_ [34]. The latter parameter entered their kinetic analysis as the time required for configurational diffusion of the unfolded chains across the transition state region of the folding reaction coordinate. This parameter represents a type of speed barrier in that it is the shortest time characterizing passage over the free energy barrier of transition state theory (TST). It was thus interpreted as providing an estimate of the prefactor for a TST expression for folding rates, as suggested by previous theoretical treatments [35,36 and references therein]. The TST prefactor for small molecule reactions, *k*_B_T/*h*, assumes impulsive atomic motion over a potential energy saddle point and is clearly too fast to be appropriate for protein folding reactions. Knowledge of the proper prefactor is necessary in order to extract free energies and entropies of activation from folding rate data. Various rate constants intrinsic to elementary events in folding have been suggested as possible prefactors, including those for the addition of another residue to a helix, ~ (100 ps)^−1^ [37], the formation of the smallest tertiary loop (N = 3), ~ (10 ns)^−1^ [31], the most probable loop formation (N ≈ 10), (0.1 – 1 μs)^−1^ [38], and the broader estimate of the unfolded conformational equilibration time in small proteins suggested above, the end-to-end contact time (N ≈ 80), (1 – 10 μs)^−1^ [39]. The overlap between the molecular timescale observed in λ repressor [34], which probably provides the most direct estimate of the diffusive barrier crossing time, and the recent estimates of the more general diffusive interchange times of unfolded conformations obtained from cyt *c* [29,30] and non-folding polypeptides [31–33] suggests that reasonable estimates of the former quantity, central to TST theory, may possibly be obtained from the latter, which is probably more accessible to experiment. This possibility would in turn support the latter quantity as a reasonable estimate of the folding prefactor.

## 5. Earliest Folding Events (*t* < 1 ms)

The nature of a rapid collapse phase in cyt *c*, typically proceeding over tens of microseconds, remains controversial [3,8,9,12,13,18,19,40–46]. In particular, whether this collapse forms a true folding intermediate, i.e., a species separated from U by a free energy barrier that is specific to the folding of proteins, or is a trivial consequence of the diffusive contraction expected for all polymers when the favorable solvation of extended chains by denaturing cosolutes is decreased in dilution experiments is a matter that has been hotly debated.

We first review below time-resolved studies, mainly using Trp59-heme fluorescence quenching, that address the existence and nature of the barrier to collapse in cyt *c*. We then turn to kinetic studies of helical secondary structure formation on the same timescale. A fundamental question in the dynamics of protein folding has been the time order in which configurational collapse, secondary structure formation, and tertiary structure formation occur. Is the formation of elements of secondary structure an initiating event that directs later folding events, as in the framework model [47,48]? Does secondary structure formation rapidly follow hydrophobic collapse, as in the formation of a molten globule-like intermediate preceding the formation of native tertiary structure [49]? Do partial elements of native secondary and tertiary structure form essentially simultaneously, as in the transition state of the nucleation condensation model [50]? The evidence in cyt *c* reviewed here mainly addresses the second question, the simultaneity of early helix formation and chain collapse.

### 5.1. Kinetic Evidence For a Free Energy Barrier to Rapid Collapse

A process of rapid collapse has been observed directly in denaturant dilution experiments with microfluidic mixing techniques and inferred from the “burst” phase of conventional Trp fluorescence stopped-flow experiments. The first high time-resolution evidence in this regard actually preceded by a couple of years the development of microsecond mixing methods. Pascher *et al.* suggested in 1996 that a 40-μs process detected by heme visible band TROA during the photo-ET-initiated folding of cyt *c*^II^ (4.6 M GuHCl, pH 7) corresponded to its collapse into a compact denatured state [2]. Although Chan *et al.* found no evidence for such an event in Trp59 fluorescence data measured after photolysis of the CO complex in roughly similar folding conditions, Winkler & Gray pointed out that the comparison was complicated by the different unfolded starting points for the two experiments [3]. The ET experiment started with a distribution of chain conformers characteristic of denatured (bis-histidinyl coordinated) cyt *c*^III^, whereas the CO photolysis experiment started with that characteristic of denatured (His18-Fe^II^-CO coordinated) cyt *c*^II^. (In addition to the additional constraint on protein backbone conformations imposed by the second heme-histidine bond, both the polypeptide chain and the heme group are positively charged in cyt *c*^III^, whereas the reduced heme is formally charge neutral.) Another issue to be considered was whether the amplitude (not reported) of the 40-μs process was large enough to be easily observed in the Trp fluorescence data of Chan *et al.* (signal to noise ratio ≈ 10).

The burst phase amplitude in stopped-flow denaturant-dilution Trp fluorescence data reported the same year by Sosnick *et al.* [13] was interpreted as evidence for a very rapid molecular collapse. However, those authors attributed this submillisecond process to the trivial compaction expected for any polymer as the interactions between polymer and solvent are reduced during denaturant dilution. That conclusion was based primarily on the observation that its amplitude was similar in non-folding fragments and the holoprotein over a range of GuHCl concentrations. The two non-folding fragments studied comprise residues 1 – 65 and 1 – 80, which contain Trp59 and the heme group but are missing the residues for at least one large element of secondary structure, the carboxy-terminal helix.

Introducing a continuous-flow capillary mixing technique with a 45-μs dead time, in 1998 Shastry and Roder used Trp59 fluorescence to follow the refolding of acid-denatured ferricytochrome *c* after a pH jump from 2 to 4.5 [41]. The observation of a collapse process with a simple exponential time course (time constant of ~ 60 μs and amplitude of 60%, relative to complete folding in 0.4 M GuHCl) was interpreted as evidence for a discrete free energy barrier separating a true compacted folding intermediate from the unfolded state, as opposed to the (barrierless) diffusive process that might be expected for simple polymer collapse. The time constant of the collapse phase decreased slightly and its amplitude decreased sharply at higher GuHCl concentrations, suggesting an equilibrium that became less favorable as the rate of back reaction increased in high denaturant. These observations were insensitive to initial heme ligation state (e.g., during rapid denaturant dilution at pH 7 with or without the addition of imidazole). A Trp59 fluorescence T-jump study with nanosecond time resolution also reached the conclusion that the microsecond collapse involved passage over a free energy barrier (τ = 90 μs, T jump = 20 → 30 °C, 1 – 2 M GuHCl, 0.5 M imidazole, pH 7) [42].

SAXS monitoring and continuous microfluidic mixing have been used to measure the average R_g_ of the chain conformers during the refolding of acid-denatured cyt *c*^III^ (pH jump = 2 → 7, 0.2 M imidazole) [51]. The extended conformations of the unfolded state collapsed to a more compact apparent R_g_ of 16 Å by the time of the first data collection window, 150 – 500 μs, and the native R_g_ of 14 Å was reached by 10 ms after mixing. The compact:extended biphasic subpopulation ratio of Shastry & Roder [41] was used to estimate an upper limit of R_g_ ≤ 18 Å for the compact subpopulation from the SAXS data observed just after collapse.

Qiu *et al.* later addressed more directly the issue of the specificity of the collapse to folding peptide sequences raised by the burst-phase amplitude data of Sosnick *et al.* with a Trp fluorescence T-jump study of the same nonfolding cyt *c*^III^ fragments [43]. They found that fragments 1 – 65 and 1 – 80 both displayed simple exponential decays similar to that of the holoprotein (τ = 8, 10, and 16 μs, respectively), implying that the free energy barrier to collapse was similar in the folding and nonfolding protein sequences. If the free energy barrier was not specific to a significant portion of the protein residue interactions found in the natively folded protein, as the results for the fragments seemed to suggest, then this conclusion raised the question: why were the collapse rates of the nonfolding sequences so slow? Simple homopolymer theories predict a rate that is a factor of ~ 10^3^ faster [43].

Gray, Winkler, and coworkers used a variety of modified cyt *c* systems in electron transfer and fluorescence energy transfer studies to probe distance distributions at early times within the folding protein [44,45,52–54]. They found that roughly similar populations of two components, characterized by extended or compact conformations, are established within the ~ 1-ms stopped-flow dead time of their GuHCl-dilution refolding experiments (extended/compact population ratio ≈ 2). The extended subensembles have a wide distance distribution, as might be expected for the more random conformations of the denatured state, whereas the compact subensembles are narrowly distributed over distances much closer to those found in the native state. These two components appear to be in rapid equilibrium (< 1 ms) with each other before folding to the native state. The existence of this discrete equilibrium implies a cooperative transition between the two subpopulations and would appear to argue against the hypothesis that compaction is due to a gradual collapse mechanism general to polymers and not specific to protein folding. The question of the sequence specificity of the submillisecond collapse process is discussed further below.

### 5.2. Submillisecond Secondary Structure Formation

Fast time-resolved studies have looked for secondary structure formation in microsecond timescale folding intermediates in cyt *c*. The existence of such structure in folding intermediates was first implied by the burst phase amplitude in stopped-flow far-UV CD studies [55]. A central question in the dynamics of protein folding has been whether protein chain collapse (solvent exclusion), which is driven by hydrophobic forces, and secondary structure formation, which reflects more sequencelocalized residue-residue interactions, occur simultaneously. The first nanosecond timescale evidence in this regard came from a (near-null ellipsometry) time-resolved circular dichroism (TRCD) spectroscopy study by Chen *et al.*, which found that 8% of helical structure (relative to the far-UV CD signal for native state formation) was formed with a time constant ≤ 0.5 μs (after photolysis of the horse heart cyt *c* CO complex in 4.6 M GuHCl, pH 6.5, 40 °C) [6]. Later nanosecond far-UV TRCD studies found that substantial helical secondary structure (20 – 35%) formed on the submicrosecond to microsecond timescale after photo-ET initiation of folding [28,56]. A heterogeneity observed in those folding kinetics, apparently arising from slow conformational diffusion of the unfolded chains, suggested that this fast folding produced near-native structure formation in a subset of chain conformers rather than partial folding throughout the bulk of sample. The time constant for fast helix formation decreased with increasing denaturant concentration, e.g., τ ≤ 0.4 μs (the instrumental time resolution) in 4.0 M GuHCl and τ = 12 μs in 2.7 M GuHCl (pH 7, 25 °C). A faster time constant at higher denaturant concentration was also observed in microsecond molecular collapse experiments, albeit with a weaker concentration dependence [41]. The rate and amplitude of this fast helix formation process was found to depend on the amino acid sequence, specifically, the presence of His33 or H26 [57,58].

A continuous-flow mixing study of cyt *c*^III^ folding monitored with far-UV CD after denaturant dilution (GuHCl jump 4.4 → 0.7 M GuHCl, pH 4.9 or pH 5.9) found that an increase of about 20% in helical secondary structure content appeared within the ~ 0.5 ms dead time of their experiment [59]. The similarity of the result with the ns TRCD results cited above for ferrocytochrome *c* folding suggests that the oxidation state of the heme does not dramatically affect the amplitude of submillisecond secondary structure formation. A pH jump (2 → 4.5) experiment using the same mixing apparatus did not detect a significant difference in secondary structure content between the acid-unfolded state and the burst-phase intermediate. However, that experiment detected an intermediate with native-like CD that was formed with a time constant, 0.48 ms, that closely overlapped the dead time of the continuous-flow mixer. Thus, the signal from the later intermediate dominated the CD observed at the first time point measured after mixing (0.4 ms) and may have obscured detection of the burst phase CD amplitude.

### 5.3. Are Rapid Collapse and Helix Formation Simultaneous?

Equilibrium data for a large number of globular proteins in their folded and partially unfolded states show a high correlation between the compactness of a given structure and the amount of ordered secondary structure it contains [60]. This equilibrium correlation suggests (but does not prove) that collapse and secondary structure formation may also be correlated kinetically. Do they indeed occur simultaneously? The kinetic results described above seem to suggest that they do. The time constants reported for the very fast collapse phase of cyt *c*^III^ range from a low value of 16 μs to a high value of 90 μs (in 1 – 2 M GuHCl) [40–43]. The time constants reported for very fast helix formation in cyt *c*^II^ range from ~ 0.5 μs to 12 μs (in 2.7 – 4 M GuHCl) [28]. There are several factors to consider in comparing these rates. First, the variation between collapse rates measured by different methods under similar conditions appears to be rather large, indicating a lot of experimental uncertainty. Second, both processes tend to proceed faster at higher denaturant concentrations. This needs to be taken into account when comparing the collapse and secondary structure rates, which were measured at generally low and high denaturant concentrations, respectively. Third, the influence of heme oxidation state on these rates, if any, is largely unknown. Fourth, kinetic data for the rapid collapse phase of histidine variants does not seem to be available for comparison with the effect on the rate of rapid helix formation noted above of mutating out misligating histidine residues [57,58]. Given these caveats, the available evidence is not inconsistent with the hypothesis that collapse and secondary structure formation generally occur simultaneously on the microsecond timescale.

Taken together, the Trp fluorescence and CD_222_ results suggest that similar subpopulations of the holoprotein and the nonfolding fragments may undergo cooperative collapse in denaturant dilution experiments to a compacted state in which disordered tertiary interactions stabilize the simultaneous formation of significant amounts of native-like helical secondary structures (although see [46] for a different view). In this scenario, the lack of the N-/C-helix tertiary interaction destabilizes the fragments and explains the lack of further folding of native-like secondary and tertiary structure. Although the cyt *c* fragments do not fold to the native state, they still retain much of the native sequence coding for secondary structure and so may still partially fold in a sequence-specific manner. The hydrophobic forces that appear to drive a steady-state fraction of the holoprotein to cooperatively condense to compact forms in the burst phase of folding may also operate in the protein fragments. In this scenario, transient tertiary interactions within the compact state could stabilize the nascent formation of significant amounts of native secondary structure. In other words, simply removing a portion of the cyt *c* sequence may not actually provide a very good model for the nonspecific collapse expected of a simple homopolymer. A test of this idea might be provided by the type of FRET-based measurements of heme-fluorophore distance distributions mentioned above that have indicated the presence of a discrete equilibrium between a well-defined compact state and an extended state in the burst phase of the holoprotein [44,45,52–54]. The application of such measurements to the equilibrium fragments 1 – 65 and 1 – 80 would provide a further test for the existence of the cooperative free energy barrier in the fragments implied by the kinetic data.

## 6. Molten Globules as Models for Folding Intermediates

Molten globules (MG), compact protein states with substantial secondary structure and fluctuating tertiary structure, have long been of interest as equilibrium models for kinetic folding intermediates [49]. However, direct comparisons of equilibrium and kinetic intermediate properties are often difficult because equilibrium molten globules are typically found in mildly denaturing conditions, whereas kinetic intermediates are observed under folding conditions. Nonetheless, kinetic folding intermediates have been identified with equilibrium molten globule forms by the results of pulsed HX experiments in relatively slowly folding proteins such as apomyoglobin and RNase H [61,62].

Sosnick *et al.* first showed that a cyt *c* molten globule state can fold very quickly [9]. They prepared a low-pH high-salt molten globule state of cyt *c*^III^ (known as the A state; pH 2, 0.5 M NaCl) and found that it folded within the 3-ms dead time of their stopped-flow pH jump (2 → 4.9 or 6.2) experiment. They interpreted this observation as suggesting that molten globules fold too quickly for an MG → N intermediate folding step to be consistent with the ~ 10-ms barrier observed in U → N folding measurements. (A conclusion that implicitly assumes the absence of a U ^→^,_←_ MG equilibrium that could give rise to a fractional steady-state population of MG.) On the other hand, Colon and Roder studied the kinetics of A state formation and concluded that its kinetic and structural properties supported the validity of this molten globule state as a model for kinetic intermediates in U → N folding [63]. In particular, stopped-flow chloride-jump experiments (KCl jump = 0 → 0.1 – 2.0 M, pH 2, 10 °C) suggested that the U → A reaction went through a series of intermediates similar to those seen in native-state folding, including a burst phase collapse (< 1 ms) and a slower phase that may be associated with N-/C-terminal helix formation.

Pabit *et al.* studied the M-CO state (a metastable molten globule formed by partial denaturant dilution of cytochrome *c*-CO complex unfolded in GuHCl) as a model of a compact, near-native state lacking native heme coordination [64]. CO photolysis in the presence of viscous cosolutes triggered an apparent folding process (monitored with time-resolved Soret absorption) with a very fast rate constant that approached a value of (10 μs)^−1^ at the lowest solvent viscosities. This solvent viscosity dependence suggested that a viscosity internal to the protein (a property of fundamental importance to the dynamics of folding that is otherwise difficult to measure) was ultimately rate limiting.

A more extensive study of the CO photolysis kinetics of this system over a range of denaturing conditions found indirect evidence for a CO-free M state that was structurally similar to M-CO, but higher in energy [65]. This molten globule state appeared to be an obligatory kinetic intermediate in the unfolding of cyt *c*^II^, as implied by a rollover of the unfolding rate chevron plot at high denaturant concentrations.

The N → M free energy value reported in [65], 9.8 kcal/mol, was identical to that measured for an equilibrium molten globule form detected previously in cyt *c*^II^ [24]. The latter form, detected by Soret MCD spectroscopy (a probe of heme coordination), reached a maximum population within the GuHCl concentration range 4 – 5 M, i.e. just below the *C*_m_ for full unfolding (see Figure 3). Their identical unfolding free energies at zero denaturant and their similarly low *m*-values (the latter corresponding to a more modest increase in residue exposure to solvent during N → M than during N → U) suggest that the equilibrium molten globule form inferred from the M-CO photolysis kinetics and that detected more directly by Soret MCD are the same state. Moreover, these states may also be related to a lowenergy unfolding intermediate identified in the native-state HX studies. Unfolding of the 70 – 85 loop foldon in this intermediate (which presumably includes unfolding of the 40 – 57 loop foldon as well) also involves loss of Met80-heme coordination and increased disorder in the nearby loop structure of the protein. A further test of this correspondence is to compare the zero-denaturant free energies. Adding the double difference between the free energies of methionine-heme and histidine-heme binding at the different oxidation states to the corresponding Fe^III^ unfolding energy of Bai *et al.*, 6.0 kcal/mol, gives a rough estimate of ~ 9 kcal/mol as the unfolding free energy of the corresponding intermediate in Fe^II^ cyt *c* [16,66]. Indeed, native-state HX measurements on cyt *c*^II^ measured a free energy of unfolding for this intermediate of 9.2 kcal/mol [67]. Comparing the Fe^II^ foldon-derived free energy value with that for the M state in cyt *c*^II^, we find that they are reasonably close, particularly given the small deviations often observed between stabilities measured by native-state HX and other methods [68]. Similarly, the free energy of the M state measured for cyt *c*^III^ (as detected by visible heme band CD, a probe of tertiary structure near the heme) is 5.7 kcal/mol, very close to the HXdetected unfolding energy of the residue 70 – 85 foldon in the oxidized protein [24]. This agreement supports the suggestion that this low energy foldon state, posited from the HX evidence to be a folding/unfolding kinetic intermediate with vanishingly small accumulation during folding, the M state inferred from the M-CO photolysis evidence to be an obligatory unfolding kinetic intermediate, and the equilibrium M state detected by heme band CD and MCD spectroscopy, may all be essentially the same state.

In contrast to the very rapid kinetics observed in the M-CO photolysis system, the folding kinetics of a ferrocytochrome *c* molten globule form prepared by partial denaturation in sodium dodecyl sulfate (SDS) appeared to be much slower, but nevertheless faster than the ~ (200 ms)^−1^ rate observed in U → N folding. The ET-triggered folding kinetics of the molten globule form were monitored with far-UV TRCD spectroscopy for [SDS] ≤ 0.65 M (well below the critical micelle concentration for this anionic surfactant) [69]. Extrapolation of the observed rates yielded a zero-denaturant time constant of ~ 1 ms. Comparison of this rate with a zero-GuHCl extrapolation of similar far-UV TRCD measurements of U → N folding rates was roughly consistent with the notion that a molten globule-like form is a rapidly folding, productive intermediate in the latter reaction.

## 7. Is the Microsecond-Timescale Collapsed Intermediate (I_C_) a Sequence-Specific Folding Intermediate?

In returning to the question of the specificity of the collapse observed in I_C_ to protein folding forces, we note that a resolution of this issue is hampered by the fact that the dynamics of chain contraction in polymers in general is not well understood (see [42,43] and references therein). Thus it is not clear how specific a phenomenon barrier-limited collapse is in the universe of polymer behaviors. Is it necessarily specific to folding-competent protein chains, as often assumed, or is it found more widely in partially folding chains, polypeptides, or in heteropolymers in general? In this regard, it is interesting to note that extended DNA chains have been induced to slowly collapse, presumably over a free energy barrier, to a discrete compacted form by the addition of cationic cosolutes [70].

The question of the sequence specificity of I_C_ was further addressed in a study that compared the far-UV CD of the nonfolding fragments with the burst phase amplitudes measured in far-UV CD stopped-flow folding measurements on holo cyt *c*^III^ over a range of GuHCl concentrations (GuHCl jump = 4.3 → 0-4 M, pH 2 or 4.9, ~ 1 ms dead time) [46]. The authors normalized the fragment 1 – 65, 1 – 80, and holoprotein CD signals to the total number of residues, 65, 80, and 104, respectively, on the assumption that the CD in all cases arose from a non-sequence specific response of the polymer to contraction. The resulting mean ellipticities per residue were similar within experimental noise across the range of denaturant concentrations studied, which was interpreted as evidence that the burst phase CD of the holoprotein arose from the same nonspecific polymer effects presumed to give rise to the small helicity observed in the fragments.

However, we note that a different interpretation may fit the CD data equally well. Because the α- helices of the native structure are distributed rather widely across the sequence, if one assumes that the CD_222_ of both the fragments and the burst-phase intermediate arose specifically from just those helical regions (N-terminal helix, residues 2 – 14; 50’s helix, residues 49 – 55; 60’s helix, residues 60 – 68; 70’s helix, residues 70 – 75; and C-terminal helix, residues 87 – 104) and calculates normalization factors based on the number of helix-specific residues retained in the fragments, the resulting factors are similar to those calculated on the basis of total residue number. Thus, the difference between the CD arising from sequence-specific and nonspecific interactions turns out to be subtler in this case than one might expect at first sight, and the issue of the specificity of the CD_222_ signal for I_C_ to bona fide native structural elements cannot be easily decided from the burst-phase data.

More structurally specific information in this regard was found in a kinetic HX study that observed residue-resolved HX protection develop in a sequence-specific manner in the compact intermediate within 2 ms of denaturant dilution-induced refolding [22]. The increase in HX protection factors corresponding to the partial formation of secondary structure in I_C_ was localized to three major α- helices of the native structure, the two terminal helices and the 60’s helix.

The contention that the decrease in Trp59 fluorescence observed after denaturant dilution is due to a small contraction of the extended chains within U is based on the R^6^ dependence of the Förster resonance energy transfer efficiency. Sosnick *et al.* estimated that only a ~ 15% decrease in average Trp59-heme distance would be required to reduce the fluorescence intensities of the unfolded chains by ~ 50% (similar to amplitudes attributed to the formation of I_C_) because of the highly nonlinear dependence on transfer distance [13] (see also [40]). However, the magnitude of the effect of denaturant concentration on R_g_ for unfolded chains does not appear to be well known. A crude estimate based on mean-field theory predictions for the length dependence of R_g_ for a polymer in solvents of different quality (e.g., taking R_g_ ~ N^1/2^ for a θ solvent, i.e. ideal random-walk polymer behavior, as representing chain behavior at high denaturant concentration, and R_g_ ~ N^1/3^ for a poor solvent as representing denaturant-free solution [71]) suggests that it could be physically possible for denaturant dilution to generate a contraction of that order of magnitude. Indeed, fluorophore distance distributions measured in unfolded cyt *c* chains seem roughly consistent with an average contraction of this size upon the dilution of GuHCl concentration by several molar units [20]. On the other hand, SAXS measurements did not detect a change in the average R_g_ of the unfolded chains over the GuHCl concentration range 3 – 5 M, as noted above [21]. Similarly, the fluorescence intensity of the unfolded chains was observed by Chan *et al.* to change by ~ 10% over the guanidinium concentration range 3.5 – 5 M, implying a Trp-heme distance contraction of only 2%, although it is not clear how much of the change in fluorescence was actually due to a change in Trp-heme distance since a similar denaturant concentration effect was observed in a nonapeptide fragment of cytochrome *c* that contained Trp59 but not the heme group [40]. While this evidence does not clearly rule out the idea that a small contraction of the unfolded chains might contribute at least in part to rapid decay of the Trp fluorescence signal upon denaturant dilution, it is currently difficult to know what the amplitude and time evolution of this process would be with the specificity needed for a more meaningful comparison with the signals observed in folding studies. In any event, given the corroborating time-resolved evidence for formation of a discrete compact intermediate from other types of spectroscopies and folding-trigger methods, it seems unlikely that the Trp59 fluorescence evidence can simply be attributed to an accidentallycoincident barrierless polymer contraction.

Considered altogether, then, the evidence reviewed here tends to suggest that formation of I_C_, the submillisecond intermediate observed in cyt *c* folding studies, is driven by sequence-specific interactions and is not the trivial consequence of the nonspecific polymer effects expected to accompany denaturant dilution. (See [19] for a different view.) The observation of a discrete free energy barrier both in the microsecond kinetics of its formation and in the discrete equilibrium between the unfolded state and the collapsed form observed after formation (before folding to the native state) implies a cooperativity not expected of a simple polymer. Although the kinetic barrier to collapse of truncated cyt *c* sequences and the holoprotein are similar, as discussed above, enough sequence-specific folding interactions may be left intact in the fragments studied to account for this similarity. The same is true for the similarity observed between the secondary structure content of the fragments and the rapid-collapse intermediate. The large effect of sequence mutations on the kinetics of helix formation observed in the ns far-UV TRCD measurements noted above is a particularly dramatic indicator of sequence-specific interactions on the microsecond timescale. Note also that this microsecond timescale formation of secondary structure was observed under constant denaturant concentration conditions, making it additionally difficult to attribute that process to simple homopolymer solvation effects.

## 8. Is I_C_ a Molten Globule?

The evidence for near-native compaction and significant amounts of secondary structure suggest a molten globule-like structure for I_C_. The thermodynamic stability of I_C_ appears to be low, lying within ~ *k*_B_T of the unfolded state at low denaturant concentrations [53,22]. This suggests that I_C_ probably does not correspond to the M state observed in equilibrium GuHCl unfolding studies, as the latter state appears to be more stable at zero denaturant. The latter conclusion is also consistent with the alternative identification suggested above of the latter state with a (non-accumulating) very late-stage folding intermediate.

Pletneva *et al.* compared FET-derived distance distributions for the burst-phase intermediate in yeast iso-1 cyt *c*^III^ and the equilibrium A state and concluded that they were significantly different [54]. Nonetheless, better equilibrium models for I_C_ might be found in molten globule forms of lower stability, such as the SDS-induced MG state [69]. In this regard, I_C_ may be more analogous to the premolten globule state that has been detected as a second, less stable and more diffuse equilibrium intermediate than the molten globule state in the four-state denaturant-induced unfolding reactions of β-lactamase and carbonic anhydrase B [72,73].

## 9. Is I_C_ on Pathway?

Since ΔG_I,U_ appears to be small and may even be positive under folding conditions, the conversion of U → I_C_ is typically not complete. This unfavorable equilibrium means that small amounts may be present before folding to the native state, which raises the question of whether it is a productive intermediate or if most folding proceeds directly from U. To answer this question, one needs to measure the microscopic I_C_-state folding rate to see if it proportionally accounts for all of the observed U → N folding rate, the latter being the quantity typically measured in kinetic studies. Such microscopic rate information is not currently available for cyt *c* folding, although the very rapid folding rates discussed above for molten globule models suggest that it is possible that I_C_ may meet this on-pathway criterion.

## 10. Implications for Folding Mechanisms

The most fundamental implication of the kinetic results presented above is that mechanistic thinking in terms of classical pathways is probably most appropriately applied to the folding kinetics of cyt *c* observed after ~ 10 μs, the time required to reach conformational equilibration of the unfolded chains [30]. While this time regime includes most of the observed kinetics, faster processes are thought to be possible, including ultrafast helix formation (~ 10 ns) and the formation of small-loop tertiary contacts thought to represent the ultimate speed limit to folding (~ 100 ns). Such ultrafast processes may proceed on multiple pathways that, being kinetically isolated by slow conformational equilibration of the unfolded chains, may present a dynamic heterogeneity that is better described by energy landscape models [29]. A caveat to this picture is that there is presently little information about the conformational diffusion rates of protein chains within compact partially-folded states. A much slower rate in such states could extend the timescale for the possible observation of nonclassical kinetic heterogeneity. However, evidence from the M-CO state of cyt *c* suggests that conformational interchange may still be rapid within compact intermediates such as I_C_ [64].

Within the classical regime, the prefactor for TST expressions of folding rates is a fundamental parameter that remains to be determined. This prefactor is closely related to the time required for passage over the TS region, which can be considered a conformational diffusion process [36]. The present experimental evidence suggests that a rough estimate of this timescale may be obtained from the conformational diffusion times of the unfolded chains, 1 – 10 μs [30,34]. For comparison, this is encouragingly close to an estimate of 100 – 500 ns obtained for the prefactor in λ repressor folding by numerically solving a formal theoretical model for conformational diffusion over the TS barrier [36].

**Figure 4.**
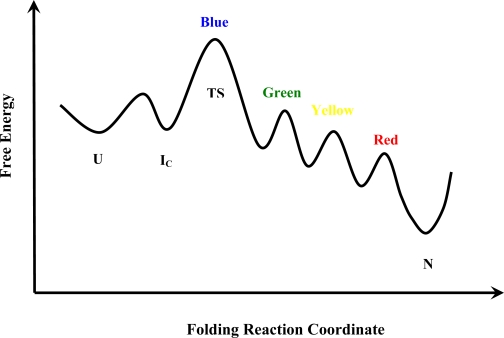
Schematic of a possible cytochrome *c* folding pathway U ⇔ I_C_ → N shown as free energy vs. reaction coordinate. The equilibrium observed for the first step implies that I_C_ lies within ~ *k*_B_T in free energy from U. Formation of the (Blue) N-/C-terminal helix foldon is the rate-limiting barrier to folding (TS). After passage over TS, the remaining foldons (Green, Yellow, and Red) are formed very rapidly through a sequence of ephemeral intermediates leading to N. (For simplicity, the foldon labels are shown by the respective energy barriers leading to their formation.).

A possible pathway for cyt *c* folding incorporating many of the experimental results discussed above is presented in Figure 4. In particular, this model incorporates the cooperative free energy barrier between U and the rapid-collapse intermediate I_C_ generally suggested by kinetic studies. Because I_C_ is not very different in free energy than U, the conversion of U to I_C_ is incomplete at steady state. The rate-limiting barrier between the steady-state mixture of U and I_C_ and its eventual conversion to N may then be presented by the formation of stabilizing tertiary contacts between the N-and C-terminal helices nascently present in I_C_. Presumably, the greater compaction and stabilization of helix formation by transient tertiary contacts in I_C_ relative to U speeds the conformational search for the TS. After the N-/C-helix barrier is passed, the undetectably rapid downhill formation of the remaining foldon units identified by Englander and coworkers leads to the native state. (Although note that the foldon-based kinetic model suggested by Englander omits I_C_ and assumes that the conformational search for the secondary structure and bihelical tertiary contacts of the TS takes place in the largely extended and nonhelical conformations of U [19].) An equilibrium molten globule state observed under mildly denaturing conditions appears to correspond closely to one of the last kinetic intermediates encountered in this downhill leg of the folding pathway, a state that conversely corresponds to an early kinetic intermediate in the unfolding pathway.

General theories of protein folding have focused mainly on the nature of the transition state, which can be probed by mutational Φ-value analysis [74]. Such studies have led to a growing consensus for a nucleation condensation mechanism involving the collapse of proteins to a compact TS state in which most of the native structural elements are partly formed [50]. An important question has been the simultaneity of collapse and secondary structure formation implied by the nucleation condensation mechanism. The apparent convergence of the nanosecond-microsecond timescale kinetic results for collapse and helix formation in cyt *c* seems consistent with this idea, albeit on a timescale that precedes the TS to native state folding. However, there is more to know about the folding pathway than the nature of the TS of the rate-limiting step. An important question is whether the shape of the free energy landscape directs the unfolded chains toward the TS, whether by biased diffusion on a funnel landscape or by the equilibrium accumulation of discrete intermediate forms on a classical pathway. One limit of the nucleation condensation mechanism is the molten globule mechanism, in which an intermediate with much of the native compactness and secondary structure of the TS first accumulates along this pathway. The apparent opportunity to observe such an intermediate in cyt *c* may provide clues to the general nature of this free-energy landscape guidance, which may also operate more generally in proteins where the folding pathway does not contain enough energetic stabilization to produce observable intermediates.

## 11. Other Cytochromes *c*

Brunori and coworkers have proposed that a similar three-state mechanism involving a partially structured intermediate may serve as a consensus folding mechanism across the broader family of cytochrome *c* proteins [75]. Their studies have focused in particular on cytochromes *c* from thermophilic bacteria such as *Thermus thermophilus*. These share the same covalently bound heme and α-helical fold as found in equine and the other mesophilic eukaryotic cytochromes *c* generally addressed by this review, but may include additional elements of secondary structure as well. They found that an increase in stability of the compact intermediate in thermophiles allowed them to obtain kinetic evidence from conventional stopped-flow Trp fluorescence measurements (T = 10 °C) that more clearly suggested an obligatory on-pathway status for this intermediate than has been possible in mesophiles [76]. Further support for such a consensus folding mechanism within the family of cytochrome *c* proteins has come from kinetic evidence for similar on-pathway compact intermediates in cytochrome *c*_552_ from the thermophilic bacterium *Hydrogenobacter thermophilus* and in the F7A mutant of cytochrome *c*_551_ from the mesophilic bacterium *Pseudomonas aeruginosa* [77,78]. The phenyl to alanine replacement in the N-terminal helix of the mesophilic protein conferred increased stability to both the intermediate and the native state, thereby switching its folding mechanism from the apparent two-state pathway of the wild type protein to a three-state pathway more similar to that observed in the thermophilic cytochromes *c*.

## 12. Conclusions

At the earliest timescales, the conformational dynamics of the unfolded chains set several speed limits to folding in cytochrome *c*. The fastest of these is the time required to form small tertiary loop contacts, ~ 100 ns, which represents perhaps the ultimate speed limit to folding. The slower equilibration of unfolded chain conformations on a timescale of several microseconds marks the onset of the classical time regime in which the high kinetic heterogeneity implied by the conformational heterogeneity of U is lost and a simpler TST description in terms of activated passage over global free energy barriers, i.e. mechanistic pathways, may become adequate. In that case, the influence of the chain dynamics on folding rates may be contained in the TST prefactor. The latter is determined by the rate at which a thermally activated reactant chain crosses the TS region by a process of conformational diffusion. That process appears to proceed on a microsecond timescale that is similar to that observed for the equilibration of chain conformers within the unfolded state.

On a (denaturant dependent) timescale of microseconds to tens of microseconds (which may overlap that for the conformational dynamics of the unfolded chains) several lines of evidence point to the rapid collapse of the chains over a small free energy barrier to a compact intermediate with native-like secondary structure. The driving force for this transition is not strong, as the free energy of I_C_ lies within ~ *k*_B_T of the unfolded state energy. Taken together, the results of time-resolved fluorescence, far-UV CD, and amide HX studies suggest that the steady-state equilibrium between U and I_C_ is established rapidly and persists until the protein folds to N. The near-native compactness afforded by the collapse to I_C_ provides (nonnative) tertiary contacts that stabilize the formation of native-like elements of secondary structure. Those elements appear to include the terminal helices, whose proper tertiary interaction is thought to be central to the rate-limiting transition state.

Summarizing the pathway for cytochrome *c* folding suggested by Englander and others, this may be viewed as a nucleation condensation mechanism in which the rate-limiting TS involves the partial formation of native tertiary contacts between the N- and C-terminal helices on the millisecond timescale. This step is then followed without observable delay by the sequential stabilization of the remaining foldon units of native secondary and tertiary structure in a framework-like manner. It is possible that the conformational search for the N-/C-terminal helix TS proceeds in the largely extended and nonhelical conformations of U. However, the early-time kinetics of cytochrome *c* suggest that collapse and the simultaneous formation of nascent secondary structure precede the ratelimiting TS in a molten globule-like extension of the nucleation condensation mechanism to include the compact intermediate state I_C_. Further experimental tests of this idea may come from kinetic studies of protein backbone and residue side chain conformational diffusion and folding rates in molten-globule model systems, the results of which may shed light on the speed of the TS conformational search within I_C_ and its possible status as an obligatory folding intermediate.
