# Correction: Goldbeck, R.A., *et al*. Early Events, Kinetic Intermediates and the Mechanism of Protein Folding in Cytochrome *c. Int. J. Mol. Sci.* 2009, *10*, 1476–1499.

**DOI:** 10.3390/ijms10041728

**Published:** 2009-04-17

**Authors:** Robert A. Goldbeck, Eefei Chen, David S. Kliger

**Affiliations:** Department of Chemistry and Biochemistry, University of California, Santa Cruz, California 95046, USA; E-Mails: chen@chemistry.ucsc.edu (E.C.); kliger@chemistry.ucsc.edu (D.K.)

By mistake, we omitted the support from the National Institutes of Health (U.S.A.) in the Acknowledgements section in our paper recently published in *Int. J. Mol. Sci.* [1]. Therefore, the Acknowledgements section is revised as follows:
